# Supplementary material for: Impact of the Deletion of Genes of the Nitrogen Metabolism on Triacylglycerol, Cardiolipin and Actinorhodin Biosynthesis in Streptomyces coelicolor
Source: Microorganisms. 2024 Jul 30;12(8):1560. doi: 10.3390/microorganisms12081560 (PMC11356632; doi:10.3390/microorganisms12081560)

Figure S1: Total lipid content, determined with FTIRS, of *S. coelicolor* M145 and derivatives of this strain deleted for the genes *glnR/sco4159*, *glnRII/sco2213*, *glnA/sco2198*, *glnII/sco2210, glnE/sco2234, nnar/sco2958,gdhA/sco4683, glnD/sco5585, glnK/sco5585, glnA2/sco2241, glnA3/sco6962, glnA4/sco1613* as well as the double mutants *afsQ1/sco4907*&*afsQ2/sco4906* and *amtB/sco5583*&*glnK/sco5584*). The strains were grown, in triplicates, on modified solid R2YE proficient in phosphate (5mM) at 28 °C for 72 h. Mean values of the three replicates are shown as histograms with error bars representing standard error (p > 0.05; Tukey-adjusted comparisons).

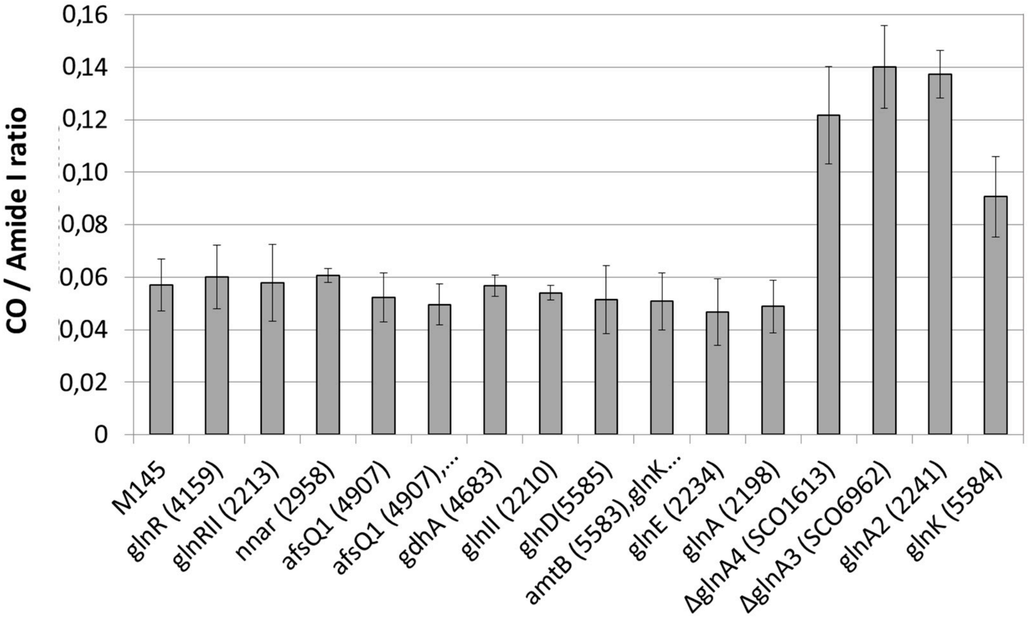

Figure S2: Total lipid content of *S. coelicolor* M145 (grey histograms), determined with FTIRS, and of a derivative of this strain deleted for the gene *pup/sco1646* (white histograms) and this mutant strain complemented by the gene *pup/sco1646* cloned into the vector pSET152 (white histograms) (1). The three strains were grown, in triplicates, in solid R2YE medium limited in phosphate (1mM), for 48 h, 72h and 96h. Mean values of the three replicates are shown as histograms with error bars representing standard error (p > 0.05; Tukey-adjusted comparisons).

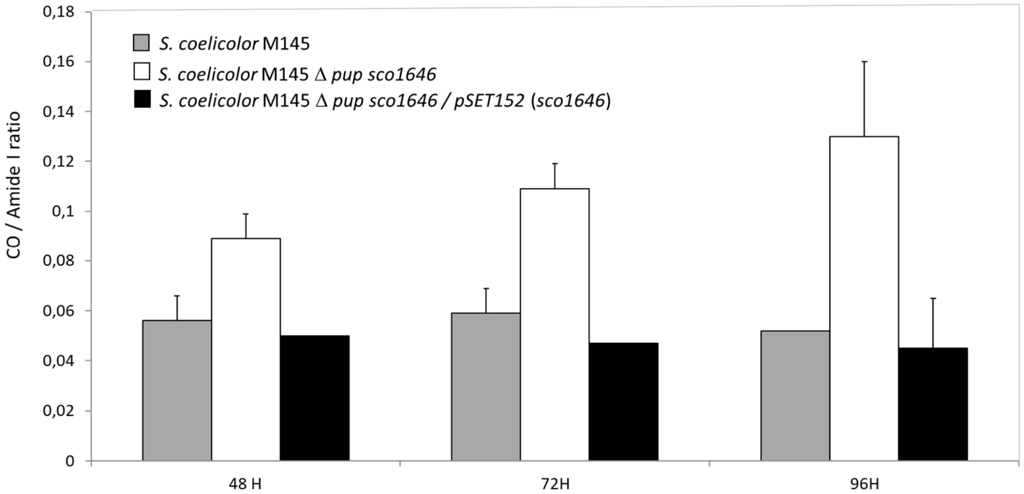

Figure S3: Total lipid content, determined with FTIRS of *S. coelicolor* M600 and derivatives of this strain deleted for *dasR*/sco*5231* and for genes of the DasR regulon involved in N-acetylglucosamine (NAG) degradation and up-take, including the genes *nagA*/*sco2758* (NAG-6-phosphate deacetylase), *nagB*/*sco5236* (glucosamine-6-phosphate deaminase/isomerase), *nagK*/*sco4285* (NAG kinase) and *nagE2*/*sco2907* (high-affinity NAG permease) (**A**) as well as of *S. coelicolor* M145 and *S. coelicolor* M600 (**B**). These strains were grown, in triplicates, on modified solid R2YE medium limited in phosphate (1mM) at 28°C for 72h. Mean values of the three replicates are shown as histograms with error bars representing standard error ($p > 0.05$; Tukey-adjusted comparisons).

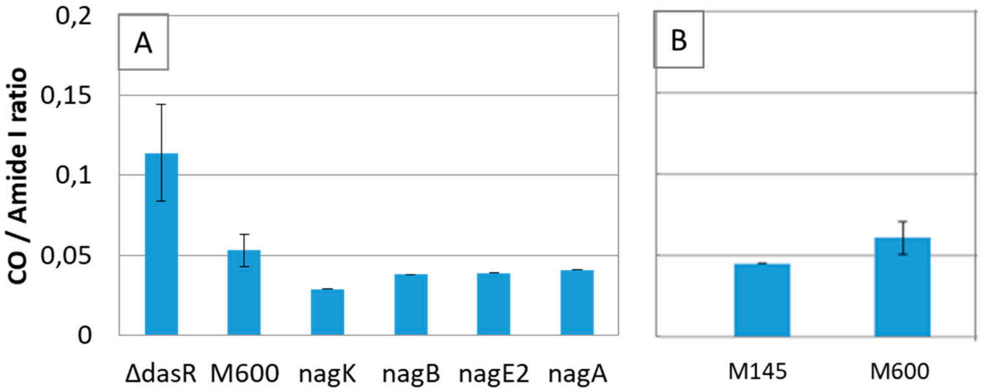

Supplement: Supplementary file 1 [file microorganisms-12-01560-s001.zip › microorganisms-3138058-supplementary.pdf]
